# Rationing of nursing care in Internal Medicine Departments—a cross-sectional study

**DOI:** 10.1186/s12912-023-01617-x

**Published:** 2023-12-04

**Authors:** Maria Jędrzejczyk, Beata Guzak, Michał Czapla, Catherine Ross, Ercole Vellone, Jan Juzwiszyn, Anna Chudiak, Mikołaj Sadowski, Izabella Uchmanowicz

**Affiliations:** 1 Department of Nursing and Obstetrics, Faculty of Health Sciences, Wroclaw Medical University, Wroclaw, Poland; 2 Center of Postgraduate Education for Nurses and Midwives, Warsaw, Poland; 3 Institute of Heart Diseases, University Hospital, Wroclaw, 50-566 Poland; 4 Department of Emergency Medical Service, Wroclaw Medical University, ul. Parkowa 34, Wroclaw, 51-616 Poland; 5 Group of Research in Care (GRUPAC), Faculty of Health Science, University of La Rioja, Logroño, 26006 Spain; 6 The Centre for Cardiovascular Health, School of Health and Social Care, Edinburgh Napier University, Edinburgh, EH11 4BN UK; 7 Department of Biomedicine and Prevention, University of Roma Tor Vergata, Rome, Italy; 8 Department of Anaesthesiology and Intensive Care, University Hospital, Wrocław, 50-556 Poland

**Keywords:** Rationing nursing care, Quality of patient care, Job satisfaction, Nurses, Perceived Implicit Rationing of Nursing Care (PIRNCA)

## Abstract

**Background:**

Implicit rationing of nursing care refers to a situation in which necessary nursing care is not performed to meet all of the patients’ needs.

**Purpose:**

To examine the factors influencing the rationing of nursing care, nurses’ assessment of the quality of patient care, and their job satisfaction in Internal Medicine Departments.

**Methods:**

A cross-sectional descriptive study was undertaken. The study included 1164 nurses working in the Internal Medicine Departments in 8 hospitals (Lower Silesia, Poland). The Perceived Implicit Rationing of Nursing Care instrument was used.

**Results:**

Respondents rarely ration nursing care, with a mean score of 1.12 (SD = 0.68). The mean score for quality of patient care was 6.99 (SD = 1.92). In contrast, the mean job satisfaction score was 6.07 points (SD = 2.22). The most important predictors of high rates of rationing of nursing care were work experience of 16–20 years (regression parameter: 0.387) and a Bachelor’s degree in nursing (regression parameter: 0.139). Nurses’ assessment of the quality of patient care ratings were increased by having a Master’s degree in nursing (regression parameter: 0.41), and significantly decreased by work experience of 16–20 years (regression parameter: -1.332). Independent predictors of job satisfaction ratings in both univariate and multivariate analysis were Master’s degree and long-shift working patterns.

**Conclusion:**

The factors that influence an increased level of nursing care rationing on medical wards are nurse seniority, exceeding 16 years and female gender. Obtaining a Master’s degree in nursing indicates improved nurses’ assessment of the quality of patient care.

## Background

Implicit rationing of nursing care is the inability of nurses to perform all patient care activities. It refers to the deliberate withholding or curtailing of essential nursing services due to a variety of factors, such as staff shortages, time constraints, insufficient resources and conflicting priorities. The issue is a growing problem in healthcare facilities around the world and can be a directly observable result of low staffing levels and poor working environments for nurses [1]. Nurses are responsible for managing the care of multiple patients during their shift, which involves the continuous prioritization of nursing care activities both within and across assigned patients. In cases where the demand for nursing care activities exceeds the available time of a nurse, nursing time becomes a limited resource that must be rationed. Rationing nursing time is essentially equivalent to rationing nursing care. Implicit rationing of nursing care is when the necessary nursing actions for patients are withheld or not carried out due to resource constraints [2]. These necessary actions encompass nursing assessments, problem identification, care planning, the implementation of various interventions: independent, interdependent, and dependent, and the evaluation of care. These activities are considered vital in the nurse’s judgment, nursing practice standards, and nursing knowledge for patients to attain their desired outcomes.

A shortage of nursing staff is the main cause of rationing of nursing care [3, 4], and this issue has been explored in various scientific studies [5–7]. Nurses need sufficient time, skill mix and level of employment to deliver quality of nursing care [8]. Where these are lacking they may not be able to deliver the necessary nursing care to all patients. They are unable to allocate the required amount of time and resources to each patient, and thus nursing rationing occurs [9, 10]. The difference between explicit and implicit rationing in nursing care primarily lies in the approach to decisions regarding resource allocation and nursing care provision. Explicit rationing of nursing care means that decisions about resource allocation and nursing care are made openly and publicly. Implicit rationing of nursing care is a situation where decisions regarding the allocation of nursing care are made in a non-transparent or non-public manner [11].

There are insufficient studies on the rationing of nursing care, nurses’ assessment of the quality of patient care and nurses’ job satisfaction in Internal Medicine Departments in Poland. In addition, previous studies included a small group [12–15]. To further explore the issue, we conducted the current study to provide additional scientific evidence on this topic.

Internal Medicine Departments are specialized medical units in hospitals or healthcare facilities that focus on diagnosing, treating and caring for patients with various diseases and disorders that do not require surgical intervention. These are the largest hospital departments in Poland (containing a minimum of 25 beds per ward). Typically, these wards house patients with moderate to severe conditions and generally are understaffed given the intensity of the work.

In Poland, there are two groups of nurses who have a license to practice. These two groups can be divided into, nurses with a secondary education who received the right to practice by way of graduating from a medical high school. These 5 year programs delivered at a medical high school is no longer available. The second group are nurses with a university education, who received the right to practice after graduating with a Bachelor’s degree in Nursing. Bachelor degree programs for nurses were established in universities in 1998 [16].

The primary aim of our study was to examine the factors influencing the implicit rationing of nursing care, nurses’ assessment of the quality of patient care, and their job satisfaction in Internal Medicine Departments.

## Methods

### Study design

A cross-sectional descriptive survey was conducted with the reference population comprised of nurses from Lower Silesia (Poland). The Strengthening the Reporting of Observational Studies in Epidemiology guidelines were followed [17].

### Participants

The study sample included 1164 nurses from Poland, Lower Silesia. The survey was conducted at each of the 8 hospitals in Wroclaw and the target population was nurses from Internal Medicine Departments. The population of nurses in Poland is approximately 300, 000, therefore the sample size was set at a minimum of 385 nurses which would enable a margin of error of ± 5% (95% confidence interval).

Inclusion criteria consisted of nurses working in the aforementioned Internal Medicine Departments and those involved in the provision of direct patient care. The study excluded nurses in management positions and those nurses working in departments other than Internal Medicine Departments.

Participation in the study was voluntary and anonymous. The participants were informed of the purpose of the research, the need to independently complete the questionnaires, their right to full anonymity, and the option to withdraw from the study at any stage.

### Data collection

The data collection was conducted from January to October 2022. Questionnaires were distributed to wards with each nurse receiving an anonymous questionnaire and a request to complete only one submission. A team of coordinators was appointed for each hospital, these individuals were responsible for the distribution of the questionnaires and collection of completed questionnaires. One thousand one hundred and sixty-four completed questionnaires were collected and included in the analysis. Questionnaires that were not properly completed or contained missing information were not included in the statistical analysis.

### Research instruments

The following tools were used for the study: a demographic data sheet, the Perceived Implicit Rationing of Nursing Care (PIRNCA) questionnaire which contained questions on nurses’ assessment of quality of patient care and job satisfaction [18].

Demographic data which was collected included information on age, sex, seniority, educational level, specialization in nursing, and working pattern. The PIRNCA was designed by Jones [18]. and is based on the original study by Schubert et al. who created BERNCA, a tool used to assess the extent of nursing care rationing [2]. The PIRNCA is a validated research instrument used to assess the perception of nurses regarding the rationing of care. It allows participants to evaluate and report their observations and experiences related to withholding or omitting necessary nursing tasks due to various factors, such as limited time, staffing, or resources. This tool provides valuable insights into how nurses perceive care rationing in their workplace and its potential impact on patient outcomes. The PIRNCA questionnaire consists of 31 questions related to the rationing of nursing care, as well as two separate questions concerning the nurses’ assessment of the quality of patient care and their job satisfaction. In the section dedicated to rationing of nursing care, respondents are asked to indicate how often they were unable to perform the listed activities during their last 7 work shifts. For each of the 31 questions in this section, the following scoring scale was adopted; never = 0, rarely = 1, sometimes = 2, often = 3. The final score is the average of points from the questions where each of the above responses was marked, with questions marked as “not applicable” excluded. Consequently, the overall score ranges from 0 to 3 and can be interpreted as higher scores indicating more frequent implicit rationing of care. Responses to questions regarding the nurses’ assessment of the quality of patient care and their job satisfaction range from 0 to 10, where higher numbers indicate that nurses have assessed the quality of patient care at a higher level and experience greater job satisfaction. The Polish version of PIRNCA, adapted by Uchmanowicz et al., confirmed the unidimensional structure and internal consistency of the Polish version of the measure [19]. The reliability of the Polish version of the tool as assessed by Cronbach’s alpha indicates high reliability (0.957).

By using both the demographic data sheet and the PIRNCA, researchers were able to gather comprehensive data on the participants’ socio-demographic characteristics and their perceptions of care rationing.

### Data analyses

The analysis of quantitative variables was conducted by calculating the mean, standard deviation, median, and quartiles. Quantitative variables were included with age and length of service expressed in years, and level of rationing of care expressed using a 3 point scale. The analysis of qualitative variables was performed by calculating the count and percentage of occurrences for each value. Qualitative variables included gender, place of residence, education level, having a specialty, and work system. Both univariate and multivariate analyses of the impact of multiple variables on the quantitative variable were carried out using a linear regression model. Predictors in our study were included age, seniority, sex, education, specialization and work system. There were no confounders in our study. The results were presented as the values of the regression model parameters with a 95% confidence interval, with a significance level of 0.05 adopted for the analysis and all *p*-values below 0.05 interpreted as indicating significant relationships. The analysis was performed using the R program (R Foundation for Statistical Computing, Vienna, Austria), version 4.1.2.

## Results

### Socio-demographic characteristics of the study group

Socio-demographic data is presented in Table 1. Approximately 1164 nurses participated in the study of which 4% were male and 96% female, with an average age of 42 years. The largest group, which accounted for 54% of participants, was composed of individuals with work experience of over 20 years, while the smallest group, 6%, comprised individuals who had less than 1 year of working experience. Regarding educational background, 77% of the participants had higher education qualifications, with 64% holding a bachelor’s degree in nursing and 13% with a master’s degree in nursing. The remaining individuals had secondary level education (23%). Over half of the respondents did not hold any nursing specialty title, while 43% had completed a nursing specialization. In terms of work schedules, 74% of the participants worked in a rotational shift system (12 h), 19% worked in a single shift system (8 h) and 7% worked in other arrangements.

**Table 1:** Socio-demographic characteristics of the study group

Parameter		Total (*N* = 1164)
Age [years]	M ± SD	42,19 ± 9,9
	median	44
	quartiles	35—49
Seniority [years]	< 1	73 (6,27%)
	1–5	142 (12,20%)
	6–10	86 (7,39%)
	11–15	89 (7,65%)
	16–20	144 (12,37%)
	> 20 lat	630 (54,12%)
Sex	Female	1117 (95,96%)
	Male	47 (4,04%)
Education level	Secondary education	267 (22,94%)
	Bachelor’s degree	743 (63,83%)
	Master’s degree	154 (13,23%)
Specialisation	no	665 (57,13%)
	yes	499 (42,87%)
Working system	Routine Shift	219 (18,81%)
	Long shift	864 (74,23%)
	< 1	81 (6,96%)

### Assessment of rationing of nursing care (PIRNCA)

The assessment of rationing of nursing care was conducted using the PIRNCA questionnaire. The average number of points obtained by the 1151 respondents was 1.12 (mean = 1.12; SD = 0.68; median = 1.1), indicating that the surveyed individuals engaged in rationing of care “rarely” The most frequently rationed nursing activities noted by the respondents were: patient education (mean = 1.72), assisting patients during walking (mean = 1.56), providing psychological support to patients (mean = 1.52), responding promptly to signaled requests (mean = 1.5), and consulting with other members of the interdisciplinary team (mean = 1.49) (Table 2).

**Table 2:** Most frequently rationed activities

Question		Never (0)	Rarely (1)	Sometimes (2)	Often (3)	Not applicable	Mean
1	Hygiene	23,97%	25,00%	23,11%	9,19%	18,73%	1,22
2	Skin care	25,69%	28,69%	21,56%	7,47%	16,58%	1,13
3	Bedding	20,10%	28,01%	23,11%	13,40%	15,38%	1,35
4	Walking assist	14,18%	24,23%	21,99%	17,87%	21,74%	1,56
5	Positions	17,61%	29,98%	24,66%	11,17%	16,58%	1,35
6	Bladder or bowel	24,40%	29,12%	21,39%	5,15%	19,93%	1,09
7	Food intake	26,89%	29,55%	19,42%	6,01%	18,13%	1,06
8	Physical comfort	27,41%	30,41%	18,47%	9,28%	14,43%	1,11
9	Medications	50,52%	26,63%	10,31%	2,49%	10,05%	0,61
10	Nutrition	45,10%	21,56%	9,62%	2,49%	21,22%	0,61
11	Wound care	37,37%	29,90%	16,32%	3,87%	12,54%	0,85
12	Intravenous port	39,78%	32,22%	15,21%	2,06%	10,74%	0,77
13	Safe practices	22,51%	26,46%	18,90%	14,78%	17,35%	1,31
14	Infections	49,14%	28,09%	11,68%	4,81%	6,27%	0,7
15	Education	13,75%	25,17%	25,34%	27,41%	8,33%	1,72
16	Preparation	31,19%	32,56%	20,62%	6,96%	8,68%	1,04
17	Emotional	16,41%	30,58%	28,18%	18,21%	6,62%	1,52
18	Physiological	43,47%	27,49%	17,01%	3,09%	8,93%	0,78
19	Behavior	22,25%	31,87%	19,85%	12,29%	13,75%	1,26
20	Safety	32,13%	31,27%	20,79%	6,44%	9,36%	1,02
21	Missed requests	26,29%	37,03%	20,36%	5,50%	10,82%	1,06
22	Waiting time	16,07%	27,23%	27,66%	15,89%	13,14%	1,5
23	Member team	16,24%	27,75%	30,76%	14,86%	10,40%	1,49
24	External unit	15,89%	24,91%	28,18%	12,71%	18,30%	1,46
25	Family member	15,46%	28,78%	30,58%	11,77%	13,40%	1,45
26	Delegations	20,53%	29,30%	26,12%	11,34%	12,71%	1,32
27	Patient data	24,74%	29,38%	24,48%	12,37%	9,02%	1,27
28	Care plan	30,33%	28,09%	22,68%	8,16%	10,74%	1,1
29	Assessment	33,68%	30,15%	19,24%	8,33%	8,59%	1,02
30	Nursing process	32,73%	27,41%	19,59%	10,65%	9,62%	1,09
31	Nursing plan	26,37%	28,78%	23,63%	10,40%	10,82%	1,2

The average assessment of the quality of patient care was 6.99 points (mean = 6,99; SD = 1.92), while the average assessment of job satisfaction was 6.07 points (mean = 6,07; SD = 2.22). This indicates that the evaluation of the quality of patient care and job satisfaction was at a moderate level. The results are presented in Table 3.

**Table 3:** Nurses’ assessment of quality of patient care and job satisfaction

PIRNCA	N	Range of values	Mean	SD	Median	Min	Max	Q1	Q3
Nurses’s assessment of quality patient care	1164	0–10	6,99	1,92	7	0	10	6	8
Assessment of job satisfaction	1164	0–10	6,07	2,22	6	0	10	5	8

The data presented in Tables 2, 3, and 4 provide an overview of the participants’ responses regarding the extent of care rationing and their assessments of the quality of patient care and job satisfaction.

**Table 4:** Relationship between the level of rationing of nursing care and socio-demographic factors—results of univariate and multivariate regression analysis

Feature		Univariate models				Multivariate model			
		**Regression parameter**	**95%CI**		**p**	**Regression parameter**	**95%CI**		**p**
Age	[years]	0,004	0,001	0,008	0,027 *	-0,006	-0,014	0,003	0,177
Seniority [years]	< 1	ref				ref			
	1–5	0,032	-0,161	0,225	0,747	0,108	-0,092	0,308	0,29
	6–10	-0,084	-0,297	0,129	0,441	0,04	-0,195	0,275	0,738
	11–15	0,085	-0,127	0,298	0,43	0,22	-0,025	0,464	0,078
	16–20	0,237	0,044	0,429	0,016 *	0,387	0,139	0,634	0,002 *
	> 20 lat	0,177	0,011	0,343	0,037 *	0,383	0,117	0,649	0,005 *
Sex	Female	ref				ref			
	Male	-0,277	-0,478	-0,077	0,007 *	-0,215	-0,418	-0,013	0,038 *
Education level	Secondary education	ref				ref			
	Bachelor’s degree	0,073	-0,022	0,168	0,132	0,139	0,032	0,246	0,011 *
	Master’s degree	-0,128	-0,263	0,007	0,064	-0,034	-0,184	0,117	0,661
Specialisation	no	ref				ref			
	yes	0,006	-0,074	0,085	0,892	-0,062	-0,149	0,024	0,157
Working system	Routine Shift	ref				ref			
	Long shift	0,013	-0,09	0,116	0,803	0,034	-0,069	0,138	0,515
	other	0,006	-0,17	0,183	0,944	0,007	-0,168	0,183	0,934

^*^ Statistically significant (*p* < 0,05)

### Relationship between the level of rationing of nursing care and socio-demographic factors—results of univariate and multivariate regression analysis

Univariate and multivariate linear regression models analysis was conducted to explore the relationship between the level of rationing of nursing care and socio-demographic data of the respondents (Table 4). In the univariate linear regression models the following were found to be the most significant predictors of rationing of nursing care: work experience of 16–20 years (regression parameter = 0.237; 95% = 0.044; CI = 0.429; *p* = 0.016) and male sex (regression parameter = -0.277; 95% = -0.478; CI = -0.077; *p* = 0.007).

The multivariate linear regression model showed that the following independent predictors were significant these included work experience of 16–20 years (regression parameter = 0.387; 95% = 0.139; CI = 0.643; *p* = 0.002), male sex (regression parameter = -0.215; 95% = -0.418; CI = -0.013; *p* = 0.038) and Bachelor’s degree in nursing (regression parameter = 0.39; 95% = 0.032; CI = 0.246; *p* = 0.011).

### Relationship between nurses’ assessment of the quality of patient care and socio-demographic factors—results of univariate and multivariate regression analysis

A univariate and multivariate linear regression models analysis of the relationship between the nursing assessment of the quality of patient care rating and socio-demographic data was conducted (Table 5). The univariate analysis, linear regression models showed that a Master’s degree in nursing was a significant predictor of patient care quality assessment (regression parameter = 0.41; 95% = 0.012; CI = 0.808; *p* = 0.044).

**Table 5:** Relationship between nurses’ assessment of patient care quality and socio-demographic factors—results of univariate and multivariate regression analysis

Feature		Univariate models				Multivariate model			
		**Regression parameter**	**95%CI**		**p**	**Regression parameter**	**95%CI**		**p**
Age	[years]	0,002	-0,011	0,014	0,819	0,033	0,003	0,062	0,03 *
Seniority [years]	< 1	ref				ref			
	1–5	-0,115	-0,768	0,538	0,729	-0,372	-1,057	0,313	0,288
	6–10	0,502	-0,276	1,281	0,206	-0,005	-0,863	0,853	0,99
	11–15	-0,106	-0,889	0,676	0,79	-0,72	-1,634	0,193	0,123
	16–20	-0,643	-1,392	0,105	0,093	-1,332	-2,275	-0,39	0,006 *
	> 20 lat	-0,127	-0,706	0,452	0,668	-1,074	-2,062	-0,086	0,034 *
Sex	Female	ref				ref			
	Male	-0,05	-0,686	0,587	0,879	-0,107	-0,756	0,542	0,747
Education level	Secondary education	ref				ref			
	Bachelor’s degree	0,007	-0,313	0,327	0,966	-0,051	-0,433	0,332	0,795
	Master’s degree	0,41	0,012	0,808	0,044 *	0,369	-0,099	0,837	0,123
Specialisation	no	ref				ref			
	yes	0,098	-0,198	0,393	0,518	0,082	-0,241	0,405	0,618
Working system	Routine shift	ref				ref			
	Long shift	-0,329	-0,703	0,044	0,084	-0,28	-0,666	0,105	0,154
	other	0,194	-0,754	1,142	0,688	0,169	-0,792	1,131	0,73

^*^ Statistically significant (*p* < 0,05)

The multivariate linear regression model showed that the most significant independent predictors of patient care quality rating are: age of the respondent (regression parameter = 0.033; 95% = 0.003; CI = 0.062; *p* = 0.03), work experience of 16–20 years (regression parameter = -1.332; 95% = -2.275; CI = -0.39; *p* = 0.006).

### Relationship between job satisfaction scores and socio-demographic factors—results of univariate and multivariate regression analysis

A univariate and multivariate linear regression models analysis of the relationship between job satisfaction ratings and socio-demographic data was also conducted (Table 6). During univariate analysis, linear regression models showed that the most significant predictors of job satisfaction ratings were: Master’s degree in nursing (regression parameter = 0.727; 95% = 0.268; CI = 1.185; *p* = 0.002), and the long-shift working system (regression parameter = -0.597; 95% = -1.028; CI = -0.166; *p* = 0.007).

**Table 6:** Relationship between job satisfaction scores and socio-demographic factors—results of univariate and multivariate regression analysis

Feature		Univariate models				Multivariate model			
		**Regression parameter**	**95%CI**		**p**	**Regression parameter**	**95%CI**		**p**
Age	[years]	-0,004	-0,018	0,011	0,636	0,057	0,023	0,09	0,001 *
Seniority [years]	< 1	ref				ref			
	1–5	-0,533	-1,288	0,222	0,167	-0,79	-1,571	-0,01	0,048 *
	6–10	-0,074	-0,975	0,827	0,872	-0,742	-1,722	0,238	0,138
	11–15	-0,164	-1,075	0,747	0,725	-1,045	-2,095	0,005	0,051
	16–20	-0,651	-1,518	0,215	0,141	-1,542	-2,618	-0,466	0,005 *
	> 20 lat	-0,662	-1,332	0,009	0,053	-1,935	-3,064	-0,807	0,001 *
Sex	Female	ref				ref			
	Male	0,896	0,163	1,629	0,017 *	0,663	-0,078	1,403	0,08
Education level	Secondary education	ref				ref			
	Bachelor’s degree	0,429	0,061	0,796	0,023 *	0,321	-0,116	0,757	0,151
	Master’s degree	0,727	0,268	1,185	0,002 *	0,702	0,167	1,236	0,01 *
Specialisation	no	ref				ref			
	yes	0,22	-0,121	0,561	0,207	0,192	-0,177	0,56	0,308
Working system	Routine shift	ref				ref			
	Long shift	-0,597	-1,028	-0,166	0,007 *	-0,453	-0,895	-0,012	0,045 *
	other	0,629	-0,436	1,694	0,247	0,525	-0,549	1,599	0,338

^*^ Statistically significant (*p* < 0,05)

The multivariate linear regression model showed that the most significant independent predictors of job satisfaction ratings were: work experience of 16–20 years (regression parameter = -.,542; 95% = -2.618; CI = -0.466; *p* = 0.005), work experience exceeding 20 years (regression parameter = -1.935; 95% = -3.064; CI = -0.807; *p* = 0.001), Master’s degree in nursing (regression parameter 0.702; 95% = 0.167; CI = 1.236; *p* = 0.01), and the long-shift working system (regression parameter = -0.453; 95% = -0.895; CI = -0.012; *p* = 0.045).

## Discussion

The study examined the factors influencing the rationing of nursing care, nurses’ assessment of the quality of patient care and their job satisfaction in Internal Medicine Departments. The most frequently rationed nursing activities were as follow: patient education, assisting patients during walking and providing psychological support to patients. Nurses’ assessment of the quality of patient care and job satisfaction was found to be at a moderate level. Work experience of 16–20 years and female gender were factors indicating the highest level of care rationing. Work experience of 16–20 years and the age of respondents reported lower patient care quality ratings, while having a Master’s degree in nursing raised patient care quality ratings. A Master’s degree in nursing increases job satisfaction, while the experience of shift work decrease it.

The number of scientific articles on implicit rationing of nursing care has significantly increased over the past decade, and several extensive reviews have shown that implicit rationing of nursing care is a problem worldwide [20]. Studies conducted globally have shown that the rationing of nursing care occurs in approximately 40% of cases [21]. Zeleníková et al. who conducted a study in four Central European countries found that the percentage of nurses who left one or more care activities unfinished was high, ranging from 95.2% (Slovakia) to 97.8% (Czech Republic), approximately 97.7% of nurses in Poland, and 97.5% of nurses in Croatia [22]. In a review conducted by Jones et al., the majority of nursing staff (55–98%) reported leaving at least one task unfinished [20]. The most frequently cited reason for nursing care rationing in the literature is staffing shortages. Hegney and colleagues, in a cross-sectional study involving nearly 2,400 nurses in Australia, found that 20% to 40% of the surveyed nurses reported being unable to provide the required care due to inadequate staffing levels and insufficient skills, resulting in excessive workloads and nursing care rationing [23].

In our study, using the PIRNCA questionnaire, it was established that nursing care rationing occurred in the participant group, and the average number of points obtained by the respondents was 1.12 (SD = 0.68), indicating that the respondents rationed care “rarely”. In the study conducted by Zeleníková et al., the results ranged from 1.13 to 1.92 (“rarely”) [22]. Other studies confirmed that emotional or psychological support for patients, failure to respond to reported requests and lack of patient education, are the most commonly omitted activities [12, 22]. Papastavrou et al. emphasized that omitting certain nursing care activities is one of the main factors influencing the quality of care [24]. Activities related to patient communication and emotional or psychosocial support disrupt the patient-nurse communication, undermine trust, and exacerbate patient isolation [25]. Szpringer et al. demonstrated the significant impact on patient satisfaction of nurse-patient ratio, respecting their dignity, and assistance with daily activities [26].

Our own research showed that the average job satisfaction score was 6.07 points (SD = 2.22), and the nurse’s assessment of the quality of patient care was rated 6.99 points (SD = 1.92). In Poland, Jarosz et al. reported in their study the average job satisfaction score was 5.95 and the nurses’ assessment of the quality of patient care was 6.88, indicating a moderate level of job satisfaction and patient care quality [27]. In another Polish study by Młynarska et al., the average job satisfaction score was 7.13 points, and the average assessment of the quality of care was 6.05 points [28]. A Slovakian study by Kalánková et al. obtained higher results than our own study with an average job satisfaction score of 7.24 points and the assessment of the quality of patient care 7.94 points, indicating a high level of care and job satisfaction [29].

The analysis conducted in our study also highlighted the influence of several socio-demographic variables on the level of rationing of nursing care. The predominant determinants of elevated care rationing levels included longer work experience and being female. Moreover, the possession of a nursing Bachelor’s degree and the respondents’ age was a somewhat less pronounced influence. Notably, older nurses exhibited a higher propensity for engaging in care rationing. These findings align with existing research, substantiating the impact of prolonged work experience, advancing age and higher educational attainment on the phenomenon of care rationing within the nursing profession. Furthermore, many scholars have underscored additional contributing factors, such as patient-to-nurse ratios and the completion of specialized training [29, 30].

Our study also revealed that the nurses’ assessment of the quality of patient care is dependent on the seniority of nurse—the higher the seniority, the lower the assessment of care quality provided. Conversely, obtaining a Master’s degree in nursing raises the quality of care provided. In other studies, no influence of socio-demographic variables on the nurses’ assessment of the quality of patient care was found [27]. However, in the study conducted by Zúñiga et al., it was proven that nurses gave higher ratings to the quality of patient care when they rationed care less [31].

The analysis conducted in our study also showed that job satisfaction is influenced by socio-demographic variables such as higher education, work shift system, seniority, age and male gender. Rotating work shifts negatively affected job satisfaction, while obtaining a master’s or bachelor’s degree in nursing positively affects it. Respondents with the longest seniority indicated much lower job satisfaction compared to those with shorter seniority. In other studies, researchers most commonly point to other factors influencing job satisfaction, frequently mentioned factors include work atmosphere, remuneration, stress, job stability and physical effort [32, 33]. In the study by Młynarska et al., no influence of socio-demographic factors on job satisfaction was found [28].

In an era of nursing shortages, which have been demonstrated to be the main cause of care rationing, this issue is now being addressed [5–7]. Current staffing shortages are mainly due to a lack of nurses in the job market, however this hasn’t always been the case. For many years, the policy of reducing nursing resources and restructuring healthcare systems, have often involved nursing staff layoffs. Nurses were sometimes regarded as an unnecessary cost [34], and their salaries kept at a low level [3]. Today, we know that the world needs an additional 9 million nurses to achieve universal health coverage by 2030 [35], and the nursing profession is ranked third in the Job Barometer as one of the most sought-after professions in the Polish job market in the healthcare industry [36].

### Study limitations

The current study has some limitations, one of which is that the average value of the nurses’ assessment of the quality of patient care and their job satisfaction may not have a strong predictive power and may potentially distort the results. Employees may be dissatisfied with their workplace conditions but still be satisfied with the care they provide to patients. This highlights the importance of considering other factors that could influence job satisfaction and the quality of patient care.

## Conclusions

Among the factors that lower the quality of nursing care, nursing care rationing should be highlighted. The factors that influence an increased level of nursing care rationing in medical wards are seniority exceeding 16 years and female gender. Obtaining a master’s degree in nursing indicates a better assessment of the quality of patient care and nurse job satisfaction. Rotating work shift systems contribute to a decrease in job satisfaction.

## Implications

Patient safety is a fundamental principle in nursing care and should always be the highest priority. However, there is a potential risk of compromising patient safety if care and its rationing are not appropriately managed. The decision to prioritize certain tasks or one patient over another can lead to missed care or delayed interventions, which may in turn negatively impact patient outcomes. It is crucial for nurses to be aware of the potential consequences of care rationing and to develop strategies to mitigate patient safety risks. Persons responsible for employment should involve nurses in the development of human resource management policies to increase their awareness of the health care environment and the delivery of patient care. It is essential for e.g. persons responsible for hospital management to develop effective employment structures and teamwork to reduce the rationing of care. In addition, nurses should be prepared to make effective decisions when faced with excessive workloads. It would be beneficial to explore additional variables, such as workplace environment, workload, organizational support, and job-related stress, to gain a comprehensive understanding of the relationship between nursing care rationing, job satisfaction, and patient care quality. By addressing these limitations and conducting further research, we can gain a more nuanced understanding of the factors influencing nursing care rationing and its impact on job satisfaction and nurses’ assessment of quality of patient care. This knowledge can then support the development of targeted interventions and strategies to improve the overall nursing care experience and patient outcomes. In summary, the gap between evidence and practice in staffing decisions in your country’s healthcare system can be attributed to a number of factors, including resource constraints and resistance to change. To move forward, it’s essential to educate decision-makers, use data, promote collaboration, pilot new approaches and advocate to ensure that evidence is translated into improved care and job satisfaction.

## Data Availability

All relevant data are included with in the manuscript document. If it is necessary, it is possible to contact the corresponding author to get additional materials.

## Abbreviations

M: Mean
PIRNCA: Perceived Implicit Rationing of Nursing Care
SD: Standard deviation
RN: Registered nurse
